# Causes and contributory factors of maternal mortality: evidence from maternal and perinatal death surveillance and response in Ogun state, Southwest Nigeria

**DOI:** 10.1186/s12884-019-2202-1

**Published:** 2019-02-11

**Authors:** Rabiatu Sageer, Eugene Kongnyuy, Wasiu Olalekan Adebimpe, Omolaso Omosehin, Elijah Ayowole Ogunsola, Bola Sanni

**Affiliations:** 1 United Nations Population Fund, Abuja, Nigeria; 2 University of Medical Sciences, Ondo, Ondo State Nigeria; 3 Ogun State Primary Health Care Board, Abeokuta, Nigeria

**Keywords:** Maternal perinatal DeathSurveillance and response, Maternal deaths, Causes, Contributory factors, Institutionalization, Ogun state

## Abstract

**Background:**

Nigeria still ranks second globally in the number of maternal deaths. Most maternal death reviews in Nigeria are isolated research based reports from a single health facility. This study determined causes and contributory factors of maternal mortality in Ogun statefollowing a periodic State-widematernal and perinatal deaths surveillance and response (MPDSR) review.

**Methods:**

We carried out a retrospective analysis of cases of maternal deaths notified (*n* = 77) and reviewed (*n* = 45) in health facilities in Ogun State from 2015 to 2016selected using total sampling method. Using the national MPDSR structured and validated data collection tools or questionnaire, collected data was extracted from existing MPDSR data base**,** andanalyzed using the Statistical Package for Social Sciences (SPSS) software 20.0. We obtained approval from the State Ministry of Health for this study.

**Results:**

Average age at maternal death was 30.8 ± 5.7 years. Haemorrhageand pre-eclampsia or eclampsia account for 43.4 and 36.9% of causes respectively. Leading contributory factors ofmaternal deaths include inadequate human resource for health, delay in seeking care, inadequate equipment, lack of ambulance transportation, and delay in referrals services. 51.1%of the women had antenatal care while a significant proportion of the women were referred from Traditional Births Attendants (TBAs) and mission houses.

**Conclusion:**

We concluded that many of the contributory factors of maternal mortality could be avoided if preventive measures were taken and adequate care available. MPDSR provides a platform for critical evidence of where the main problems lie, and can provide valuable information on strategies which maternal mortality prevention programs should focus on. The implementation and institutionalization of MPDSR programme is on course in Ogun State. MPDSR is feasible and should be institutionalized in all states of Nigeria. A commitment to act upon the findings of MPDSR is a key prerequisite for success.

## Background

The widest disparity in health statistics compiled by the World Health Organization between developed and developing countries occurred in the area of maternal mortality, with the developing countries contributing most of the figures. At the close of the Millennium Development Goals (MDGs), Nigeria like many other countries in Sub-Saharan Africa had not only failed to achieve the goals, but still hadhigh maternal and perinatal morbidity and mortality rates. Globally,thousands of women die annually from complications during pregnancy, childbirth, or postpartum period, with most deaths occurring in developing countries [1]. These trends over the past decades had been adjudged as unacceptable, as it remained a problem of public health importance necessitating the attention of all stakeholders in maternal and child health care.

According to the World Health Organization (WHO), Nigeria had the second highest number of annual maternal deaths in the world in 2010 and contributed 14% of all maternal deaths globally [2]. Nigeria has a maternal mortality ratio of about 814 per 100,000 live births as at 2015 [3]. Within Nigeria, Maternal Mortality Rate (MMR) figures differs between geo-political zones, with southwestern Nigeria having one of the lowest rates of preventable Maternal and Perinatal deaths according to the National Demographic and Health Survey (NDHS) data [4].

Many developing countries carry out spectrum and modeling projection analysis in order to determine levels and trends in maternal mortality, due to lack of complete and reliable data in these countries. As a result, maternal mortality ratios are generated periodically and reflect situations few years prior to the surveys. Most reviews on causes and contributory factors on maternal deaths in Nigeria are isolated research-based reports from a single health facility and which may not be generalizable on large populations. As these statistics do not present national maternal mortality ratios, reviews are needed for determining trends over a period of timeso asto develop policies for reducing maternal and perinatal mortality ratios by improving the quality of prenatal and obstetric care.

From 2009, Ogun and some other states in Nigeria have been involved in various pilot schemes to establish Maternal Death Review (MDR) processes with support from development partners. A renewed effort at a comprehensive approach that included perinatal and integrated surveillance and response components into this approach was approved by the Nigeria National Council on Health in 2013. It mandated all states to implement a comprehensive Maternal and Perinatal Death Surveillance and Response (MPDSR). By implementing MPDSR review, it is possible to obtain critical evidence of where the main problems lie, which need to be addressed to reduce maternal deaths. This study determined causes and contributory factors to maternal and perinatal mortality in Ogun state in Southwestern Nigeriafollowing a periodic State-wide MPDSR review.

## Methods

### Study area

The study area was Ogun State in Southwestern Nigeria. The State hasan estimatedtotal population of 6,084,327 (with 3,078,669 males and 3,005,657females) derived from projections based on a 3.3% annual growth rate of the 2006 National Population Census figure for 2016. There has been a notable rise in the population and especially that of women of Reproductive Age (WRA) to an estimated 1,366,207 [5]. We obtained approval from the State Ministry of Health for this study.

Anecdotal review and analysis of 2007 to 2009 figures for Ogun Stateshowed a State level Maternal Mortality Ratio of 179 per 100,000 live births and Infant Mortality Ratio, Perinatal Mortality Ratio and Under-5 Mortality Ratio of 69/1000, 21/1000 and 27/1000 live births respectively. Modern contraceptive prevalence rate was 21.5 and 26.0% for all methods. Though most deliveries take place at health facilities (30.7% public and 44% private), the percentage of Traditional Birth Attendants (TBA)/Home delivery is still significant (24.8%) and in most situations,cases are often referred from this source to health facilities when it is already too late.

### The study population

Constitutes all cases of maternal deaths that occurred in health facilities in Ogun Statein 2015 and 2016. The inclusion criterion is that such death should have been notified by the health facility where death occurred to the State MPDSR system which is a part of the national response.

### Study design and scope of reviews

The study which was retrospective in design,covered an up-to-date epidemiological and programmatic review of maternal deaths data emanating from the MPDSR State response in 2015 and 2016. Quality, consistency, completeness and availability of data, and the investments needed to improve measurement of trends were also assessed. Observed trends in the surveillance and response systems were also assessed. Other activities carried out was a comprehensive desk review of documents, policies, guidelines, and reports, related to maternal mortality in Ogun State producedtill date.

### Sampling methodology

A total sampling of all the MPDSR supported health facilities was carried out. In addition, a total sampling of all deaths notified within the period was made.

### Research instruments

The national MPDSR tool (semi-structured, pretested and validated data collection questionnaire) was used by all the facilities for reporting or notification of deaths, and timely review of the same by designated facility staff. The existingStatedata-base was updated and validated by visiting some of the facilities at random and checking for consistency and availability of data.

### MPDSR data collection methods

The State MPDSR response team went round the supported healthfacilities to ensure that no routine notification andreview forms were left behind, and that allforms got to the State Monitoring and Evaluation unit where the MPDSR database was being regularly updated. The State MPDSR data-base was a compendium of the responses in the data collection tool per Local Government Area (LGA) and per health facility. Secondary data analysis methodology only was deployed. MPDSR implementation efforts at health facility level were also assessed. A review of presentations made by the health facilities at the annual review meeting was also done.

### Study variables

These include pattern of notification and review of maternal deaths, socio-demographic characteristics and some reproductive health history of the women who died within the period, includingthe causes and contributory factors to maternal mortality.

### Ethical approval

Ethical Approval was obtained from Ogun state primary health care board ethical review committee. Written informed permission was obtained from the Medical directors of selected health facilities and the Executive Secretary of the Primary Health Care Board to use data.

### Data analysis

The SPSS software 20.0 was used in analyzing the data. The validity of data entered was ensured by double entry and random checks. Uni-variate analysis was presented using frequency tables and charts. Trends in deaths notification and review were also analysedusing charts.

## Results

**Fig. 1:**
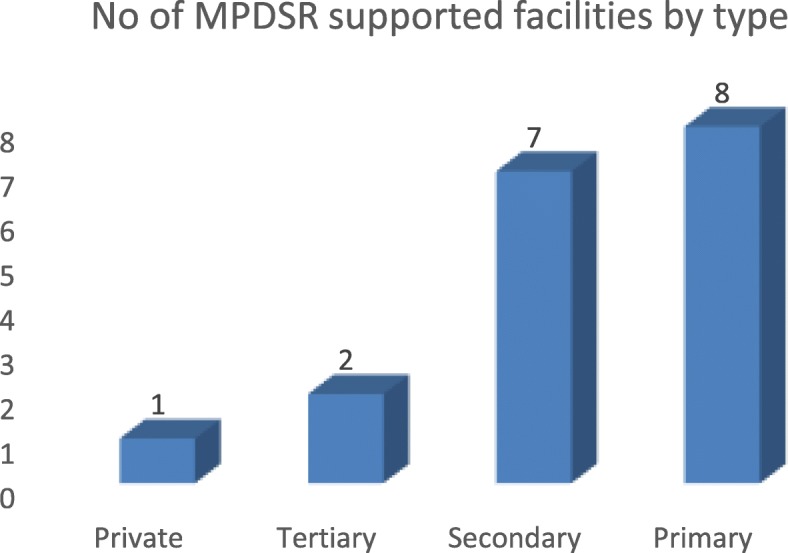
Distribution of MPDSR Sites by Type of Facility

**Fig. 2:**
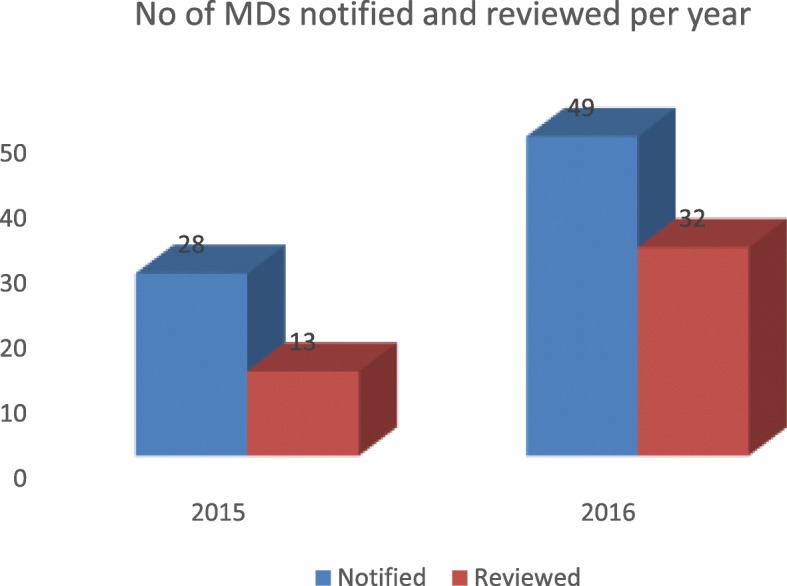
Number of Maternal Deaths Notified and Reviewed by Year

Figure 1 shows the distribution of facilities by type. Eight (44.4%) of the facilities were Primary Health Care centers, 7(38.9%) were secondary or general hospitals, 2(11.1%) were tertiary or teaching hospital, while only one (5.6%)of them was a private health facility. This shows a scale up from 12 health facilities in 2015, to 18 health facilities in 2016. In 2015, only 13 (46.2%) of the 28 maternal deaths notified were reviewed. In 2016, thirty two (65.3%) of 49 cases notified were reviewedas shown in Fig. 2.

Table 1 shows the socio-demographic characteristics of the women whose death were both notified and reviewed during the period. Average age at death was 30.8 ± 5.7 years, 41(91.9%) of them were married, 31(68.9%) live in urban areas, about 8(17.8%) were of primary, even secondary level education 4(8.9%), while 21(46.6%) belonged to the middle socio-economic class by occupation such as artisans and trading.

**Table 1:** Socio-demographic profile of (reviewed) women who died (*n* = 45)

Variable	N	%
Age (Mean 30.8 ± 5.7, years)		
15–24	4	8.9
25–34	26	57.8
35–44	15	33.3
Marital status		
Married	41	91.9
Not married	4	8.9
Residence		
Urban	31	68.9
Rural	14	31.1
Education level		
Primary	8	17.8
Secondary	8	17.8
Tertiary	8	17.8
No formal	6	13.3
Not recorded	15	33.3
Occupation		
High	8	17.8
Middle	21	46.6
Low	4	8.9
No response	12	26.7
Religion		
Christianity	28	62.2
Islam	11	24.4
Others	6	13.3

**Fig. 3:**
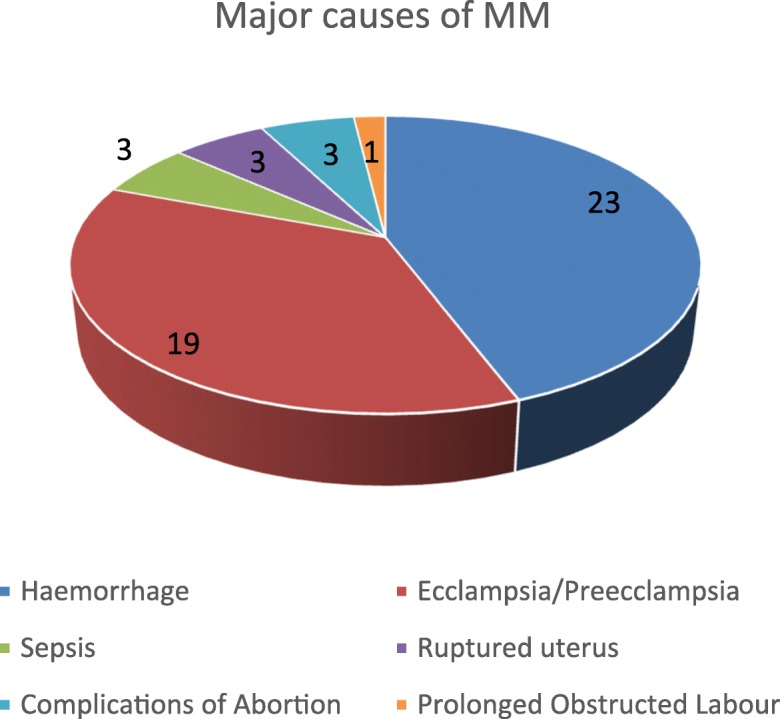
Frequency of the Major Causes of Maternal Mortality

The six leading or major or direct causes of maternal mortality in Ogun State are shown in Fig. 3. Haemorrhage accounts for 43.4% of causes, while pre-eclampsia or eclampsia accounts for 36.0% of deaths. Others causes include septicaemia, ruptured uterus, and complications of unsafe abortions (each accounting for 5.7% of causes).

Table 2 showed that a little less than one fifth of the women had Ante Natal Care whatever is the source. The time interval between admission and death showed that about a little less than a proportion of one third each (28.9 and 31.1%) of the women died either within the first 6 h or a period after 24 h after admission. Table 2 showed the sources of women who were admitted or the sources of referrals and the (previous) care providers at the referral sites where death eventually occurred. The largest proportion was from another or the same health facility. The TBAs and Mission houses provided care to 6(13.3) and 2(4.4%) of women respectively, while the home provided a base to 10(22.2%) of women and serving as route of out-referrals. The period of death of the pregnant women reviewed showed that the puerperium accounts for the period during which 25(56.8%) of deaths occurred (most likely suggesting Post Partum-Haemorrhage as a significant likely cause of deaths in most of the women).

**Table 2:** Obstetrics/Gynecological history of the (reviewed) women who died (n = 45)

Variable	F	%
Received ANC as at last pregnancy		
Yes	23	51.1
No	8	15.6
Not sure/don’t know	15	33.3
Sources of admission/initial care		
Other health facilities	26	57.8
TBAs	6	13.3
Home	10	22.3
Mission houses	2	4.4
others	1	2.2
Period of maternal deaths		
Post-partum	25	55.6
Labour/delivery	7	15.6
3rd trimester ANC	6	13.3
2nd trimester ANC	6	13.3
1st trimester ANC	1	2.2
Time interval between admission and deaths		
< 6 h	13	28.9
6–12 h	3	6.7
12–24 h	7	15.6
> 24 h	14	31.1
Not indicated	8	15.6

The leading contributory factors or non-medical causes of Maternal and Perinatal Deaths as shown in Table 3, and these include inadequate manpower (21.6%), delay in seeking help(11.8%), lack of essential equipment/medications/blood(7.8%), lack of ambulance/transportation(15.7%), delay in referrals most especially of high risk pregnant women(11.8%), poverty/lack of money (9.8%) and lack of awareness of danger signs by care givers(5.9%).

**Table 3:** Contributory factors/Non-medical causes of Maternal and perinatal deaths

Contributory factors (*n* = 51)	n	%
Manpower shortage	11	21.6
Lack of transport/ambulance	8	15.7
Delay in seeking care	6	11.8
Delay in referrals	6	11.8
Lack of resuscitative skills/efforts	5	9.8
Poverty	5	9.8
Lack of equipments/medications/blood	4	7.8
Failure to recognize danger signs	3	5.9
Inadequate power supply	3	5.9

Going by the total number of life births in each institution or health facility, Table 4 shows the institutional Maternal Mortality Ratio (iMMR) per health facility and LGA on the long run. The table also showed that iMMR was higher than the average national figure in all the facilities except in Oba Ademola Maternity Hospital, testifying to the unacceptably high maternal mortality indices in Nigeria. Table 4 also showed that haemorrhagehas the highest proportional mortality rate of 0.80, followed by eclampsia (0.66), with Abeokuta South LGA having the highest mortality ratio as expected. In response to the reported surveillance systems, the State team carried out capacity building for staff and stepped up monitoring and supervisory visits among others. Each facility carried a number of site specific improvement actions to address the causes and contributory factors such as provision of anti-shock garments, improved blood banking systems etc.

**Table 4:** The Institutional Maternal Mortality Ratio (iMMR) per LGA and The Proportionate risk (PR) of dying from the common causes

LGA	Facility type	No of Live births	No of maternal deaths	iMMR/10
Abeokuta South	Private	1915	21	1096
Abeokuta South	Secondary	1816	10	560
Abeokuta South	Tertiary	941	13	1381
Ifo	Secondary	496	12	2620
Ijebu ode	Secondary	1124	9	841
Ado Odo Ota	Secondary	735	6	816
Ipokia	Secondary	280	5	1785
Proportionate risk (PR) of dying from the common causes (0.5 baseline)				
Risk of dying from		Risk from cause	All causes	PR
Haemorrhage		12	15	0.80
Eclampsia/Pre-eclampsia		10	15	0.66
Sepsis		7	15	0.46
Surgery/anaesthetic complications		3	15	0.20
Ruptured uterus		2	15	0.13
Abortion		2	15	0.13

## Discussion

Institutionalizing MPDSR in Ogun State has presented the pattern and contributory factors to preventable maternal deaths in the State. The major or leading causes of maternaldeath reported (haemorrhage followed by preeclampsia and eclampsia) in this assessment had been the trend in Nigeria and other developing countries in the recent times [6–9]. The proportion of prolonged obstructed labourdue to Cephalo-pelvic disproportion (CPD) was 1.9%; far lower than the national average. This may be attributed to the prompt and easy availability of Caesarian Section services in Ogun state as part of the State response. Overall, there is overwhelming evidence from MPDSR facilities case summary reports that most maternal deaths are a fall out of un-booked cases and delay in referral from Primary Health Care centers, mission houses and or Traditional Birth Attendants (TBAs) to secondary health facilities. When a pregnant woman is not booked for antenatal care or does not deliver in a health facility, she misses all the opportunities offered by antenatal care, benefits of active management of labour, Emergency Obstetrics Care (EOC) and management of the puerperal period, thereby having a higher risk of maternal death [10]. All these opportunities are available whenskilledand equipped birth attendants take charge of the pregnancy and childbirth periods, evidently in registered and orthodox hospitals.

While some of the major causesof maternal deaths are amenable to prevention such as hypertension, the vast majority of the contributory factors to maternal death falls in the same category and include social factors such as low socio-economic status, poverty, ignorance, socio-cultural barriers to accessing antenatal care, poor personal attitude to health care, self-risk perception, transportation logistics, and delay in making referrals from other health facilities such as primary health care centers, TBAs, homes and mission houses. The solution to many of these contributory factors lies in appropriatepolicies, improved budgetary allocation to health care, improved donor support, and capacity building of Health Care Workers (HCWs). However, a good number of these factors fall outside the confines of the health care facilities.

The institutional Maternal Mortality Ratio (iMMR) is high across all LGAs where deaths were recorded and highlights the need to pay more attention tostrengthening health systems around maternal and child health care in Nigeria. In the same vein, the order of risk at a reference value of 0.5 showed that the proportionate risk of dying from Haemorrhage is highest (0.80 or 80%) followed by that of eclampsia or pre-eclampsia (0.66 or 66%). This is similar to findings from another study [11]. Though complication of anesthesia and surgery may not be categorized among the major causes of death, they are significantly important among the indirect causes, and the risk is also significantly high (0.20). This trend should serve as warning signal to care providers that more serious efforts should be put into the care of women who come down with these diagnosis. In addition, hospital management and policy makers should ensure that facilities and material resources to manage these cases should be made available and accessible at all times.

A significant proportion of the maternal deaths had the TBA, home and mission houses as their source of admission or referrals or initial care. The home is not an ideal place for antenatal care (ANC) and delivery of a baby because skilled birth attendants are absent and focused ANC and active labourmanagement is lacking [12]. Delivery at home could be associated with other social contributory factors such as poverty or low socio-economic factors, ignorance, poor attitude to ANC and several others constituting delays towards maternal mortality. Of significance are the TBAs who are usually traditionally accepted for providing maternal and child care at community level, but who are still not skilled birth attendants, not practicing Focused Antenatal Care (FANC) and whose care may be regarded as substandard for their lack of formal training in maternal and child health care. It is thus important for Ogun State to develop and operationalize a state framework for the monitoring and supervision of care of pregnant women being delivered by TBAs, most especially recognition and prompt referrals when problems are foreseen.

A closer look at the obstetrics and gynecological history showed that problems usually starts around the 2nd to third trimester of pregnancy (probably due to antepartum haemorrhage), yet the period around delivery and few hours or days after delivery accounts for most of the deaths. It is thus important to stress the need for active management of labor and the immediate post -partum period to all care providers. The concept of essential obstetrics care should be taken as the standard whether there would be complications or not. The need for prompt referrals should be stressed to health care workers in all primary health care facilities, mission houses and the TBAs so as to prevent further maternal deaths.

The low notification and review figures in 2015 may not be unconnected with the fact that MPDSR programmes could not commence in Ogun State until after the 1st quarter of the same year. The highly appreciable review rate compared to notification rate in 2015 suggested that MPDRS programmes has fully taken off in Ogun State and that the support given by the MPDSR team and donor agencies to the State health systems served as positive encouragement towards developing a culture of reporting and review of maternal and perinatal death at the health facility level. The 2016 review was a drastic improvement in the notification and review of 2015.

Several efforts were put in place by stakeholders in MPDSR response in Ogun State coordinated by the state primary health care board confirms plans to institutionalize the programmeand response in Ogun State, even after the withdrawal of donor support. In a supportive Ugandan study, the factors that influenced conduct of MPDR were existence of MPDR committees (*p <* 0.001), attendance of review meetings *(p* < 0.001) [13]. In some other studies, holding review meetings, establishment of review committees and provision of relevant basic equipmentswere motivators for taking part in MPDSR [14, 15].

## Conclusion

We concluded thatthe majority of causes and contributory factors to reported maternal deaths are preventable through combined safe motherhood strategies of focused antenatal care, prompt referral, active management of labour and immediate post-partum period and access to family planning. However, the pattern of the causes is gradually changing with deaths due to cephalo-pelvic disproportion on the decline due to availability of caesarean section services. MPDSR provides a platform for critical evidence of where the main problems lie. MPDSR also provides evidence-based recommendations to maternal health stakeholders on strategies that could significantly reduce maternal mortality. The implementation and institutionalization of MPDSR programmesis on course in Ogun State. MPDSR is feasible and should be institutionalized in all states of Nigeria. A commitment to act upon the findings of MPDSR is a key prerequisite for success.

## Abbreviations

CPD: Cephalo-Pelvic Disproportion
EOC: Emergency Obstetrics Care
FANC: Focused Antenatal Care
HCW: Health Care Workers
iMMR: Institutional Maternal Mortality Ratio
LGA: Local Government Area
MDG: Millennium Development Goals
MDR: Maternal Death Review
MMR: Maternal Mortality Rate
MPDSR: Maternal, Perinatal Deaths Surveillance and Response
NDHS: National Demographic and Health Survey
TBAs: Traditional Births Attendants
WHO: World Health Organization
WRA: Women of Reproductive Age
